# Gel Dosimetry

**DOI:** 10.3390/gels9040311

**Published:** 2023-04-07

**Authors:** Salvatore Gallo, Silvia Locarno

**Affiliations:** 1 Dipartimento di Fisica “Aldo Pontremoli”, Università di Milano, 20133 Milano, Italy; 2 Istituto Nazionale di Fisica Nucleare (INFN), Sezione di Milano, 20133 Milano, Italy

## 1. Introduction

The purpose of radiation therapy (RT) is to cover tumor tissue homogeneously with a planned dose while minimizing the dose to the surrounding healthy tissue [1]. At present, applied techniques in RT can deliver a dose distribution with a high conformity and precision to the treatment target volume. It is noteworthy that any error or inaccuracy in dose delivery during irradiation in these modern techniques can result in either an insufficient dose to the tumor tissue or a high dose to the adjacent healthy tissue. Hence, the determination of the 3D dose distribution in a tissue-equivalent material, before radiotherapy, can decrease any potential error. Owing to this, developing high-performance systems capable of coping with the challenging requirements of modern ionizing radiation is a key issue to overcome the limitations of 1D and 2D conventional dosimeters [2].

In this scenario, “Gel dosimetry” is the most promising tools for the evaluation of 3D high-spatial-resolution dose distributions, and the studies regarding these materials represent the starting point for developing performance and innovative systems [3,4]. “Gel dosimeters” are based on chemical dosimeters in which radiation-induced chemical reactions occur. The addition of gelling agents reduces the diffusion of chemical reaction products and, consequently, the dosimeter is spatially stabilized. Gel dosimeters generally consist of two types: Fricke gels (FG) [5] and polymer gels [6]. In the first one, ferrous ions (Fe^2+^) in ferrous sulphate solutions are dispersed throughout the gel matrix, while in the second one, monomers (such as acrylamide), are dispersed in the matrix. The radio-inducted variations can be read out by magnetic resonance imaging (MRI), x-ray computed tomography (CT), optical scanning, and ultrasonography. Despite extensive research in recent decades, gel dosimeters have yet to achieve widespread clinical acceptance, mainly because of three major practical concerns: the toxicity of active materials, oxygen sensitivity of the dose response, and the spatial instability of dose information.

A number of gel dosimetry systems have been developed over the years with differing mechanisms of operation and varying degrees of success in alleviating the practical issues, inhibiting a clinical application.

This current Special Issue is a thorough collection of articles dealing with the synthesis and characterization of hydrogels, of which the authors show the mechanisms of action and prospective applications for 3D dosimetry. The overview presented in this Special Issue would not be complete without mentioning novel approaches for the characterization and modeling of hydrogels for medical applications.

With this in mind, the goal of this Special Issue of *Gels*, belonging to the “Gel Analysis and Characterization” Section and titled “Gel Dosimetry”, was to collect original research manuscripts that describe cutting-edge developments in hydrogel-based materials for dosimetry and their translational applications, as well as reviews providing updates on the latest advancements in this field.

A series of manuscripts have been submitted to the Special Issue, and 11 of them have been accepted for publication. The final collection includes eight original research manuscripts and three reviews by authors from ten different countries. The contents of the published manuscripts are briefly summarized below.

## 2. Contributions

In the article of *Zirone* et al. [7], entitled “HyperArcTM dosimetric validation for multiple targets using ionization chamber and RT-100 polymer gel”, a dosimetric validation of the HyperArcTM technique, through end-to-end tests on five multi-target single-isocenter SRS plans, was carried out. The tests were performed using an ionization chamber and a commercial polymeric gel (RTgel-100) to obtain point measurements and 2D and 3D distribution of the delivered dose. The same anthropomorphic phantom was used for end-to-end testing with polymer gels. In this case, the comparison between the two distributions was performed through dose profiles and through 2D and 3D gamma index passing rate analysis relative to the various lesions. The information obtained from both the ionization chamber and polymer gel measurements confirmed that the use of a single isocenter for multiple lesions reduces the treatment time without compromising accuracy, even in the case of target volumes that are quite distant from the isocenter.

In the article of *Merkis* et al. [8], a unique measurement system and methodology was proposed that includes the development of the hardware which allows for in situ dynamic measurements of the optical characteristics of irradiated “nMAG Dose Gel” during and after the irradiation procedure. The developed system can collect data for a practically unlimited period of time. The authors found that changes in the optical properties in irradiated nMAG dosimetric gel can be detected up to 6 h after the start of irradiation. This time is significantly shorter compared to the 24 h recommendation provided by the literature. The determination of the significantly shorter time interval (compared to 24 h post-exposure recommendations) for dose gel readout and the evaluation of factors contributing to the polymerization dynamics of nMAG dose gels facilitates a further investigation of dosimetric gels and their introduction into the medical environment.

The manuscript entitled “Three-Dimensional Dosimetry by Optical-CT and Radiochromic Gel Dosimeter of a Multiple Isocenter Craniospinal Radiation Therapy Procedure” by *de Silveira* et al. [9] proposed the use of a modified radiochromic FG dosimeters in craniospinal irradiation (CSI) scenario. The found results show that gel dosimetry using the radiochromic gel combined with optical computed tomography allows a three-dimensional dose determination of a CSI procedure with complex multiple-field planning.

*Soliman* et al. [10], using UV-Vis spectrophotometry, investigated the dosimetric characteristics of hydrogel dosimeters based on polyacrylamide (PAC) as a capping agent incorporating silver nitrate as a radiation-sensitive material up to 100 Gy. Glycerol was used in the hydrogel matrix to promote the dosimetric response and increase the radiation sensitivity. Upon exposing the PAC hydrogel to γ-ray, it exhibits a surface plasmon resonance (SPR) band at 453 nm, and its intensity increases linearly with absorbed doses. The obtained results support the validity of using this hydrogel as a dosimeter to verify radiotherapy techniques and dose monitoring during blood irradiation.

The manuscript of *Scotti* et al. [11] aims to investigate, by means of spectrophotometric analyses, how the sensitivity to the radiation dose and the range of the linearity of the dose–response curve of Fricke gel dosimeters based on poly(vinyl alcohol) (PVA) as the gelling agent [12] and glutaraldehyde (GTA) as the cross-linker and loaded with xylenol orange (XO) [13] dosimeters are influenced by FAS and XO concentrations. The effect of different concentrations of such compounds on self-oxidation phenomena occurring in the investigated Fricke gel dosimeters was evaluated.

In the manuscript of *Toyohara* et al. [14], radioactivity was measured in a micellar gel dosimeter, a polymer gel dosimeter, and water by carbon ion beams irradiation at various beam energy conditions. Monte Carlo simulation was also performed to estimate the radioactivity. Short-lived positron-emitting nuclides were observed immediately after irradiation, but they decayed rapidly into the background.

The manuscript of *Rabaeh* et al. [15], entitled “Improved Dose Response of N-(hydroxymethyl)acrylamide Gel Dosimeter with Calcium Chloride for Radiotherapy”, deals with the impact of calcium chloride (CaCl_2_) on the performance of N-(hydroxymethyl)acrylamide (NHMA) polymer gel dosimeter. The dosimeter was exposed to doses of up to 10 Gy with a radiation beam-energy of 10 MV, and the relaxation rate (R_2_) parameter was utilized to explore the performance of irradiated gels.

In the study of *Mizukami* et al. [16], the authors investigated the whole 3D dosimetry of carbon ion beam gel dosimetry by applying a new rapid MR imaging method. The rapid and accurate measurement of the T_1_ relaxation time also remains an important goal in clinical examinations. Low-noise, high-resolution 3D mapping of T_1_ relaxation times could not be achieved in a clinically acceptable time frame (<30 min). The authors also demonstrated that the whole three-dimensional dose distribution could be roughly evaluated within the conventional imaging time (20 min) and the quality of one cross-section.

Our Special Issue also covers some high-quality review articles that complement the recent literature regarding 3D gel dosimetry [17,18,19].

In the review article entitled “Recent Advances in Hydrogel-Based Sensors Responding to Ionizing Radiation” [20], the authors catalog hydrogel-based dosimeters such as polymer-, Fricke-, radio-chromic-, radio-fluorescence-, and NPs-embedded dosimeters. Most of them demonstrate a desirable linear response and sensitivity regardless of the energy and dose rate of ionizing radiation.

The review article entitled “Radiation Dosimetry by Use of Radiosensitive Hydrogels and Polymers: Mechanisms, State-of-the-Art and Perspective from 3D to 4D” by De Deene [21] provides an overview of the methods and state-of-the-art technology of 3D radiation dosimetry with gel and polymer systems.

The review article entitled “Chemical Overview of Gel Dosimetry Systems: A Comprehensive Review” by *Macchione* et al. [22] aims to contribute thorough descriptions of the chemical processes and interactions that condition the gel dosimetry outputs of ten phenomenologically addressed and particular formulations reported since 2017.

## 3. Conclusions

In conclusion, we were very pleased to guest edit this Special Issue, as it collects relevant contributions that reflect the increasingly widespread interest in hydrogels and related applications in the field of “3D Dosimetry” and “Radiation Therapy”. We hope that this Special Issue can reach the widest possible audience in the scientific community and contribute to further boosting scientific and technological advances in the intriguing world of hydrogels as well as their multidisciplinary applications. Finally, we wish for this Special Issue to help its readers to conceive both new and improved ideas about “Gel Dosimetry” in their respective fields.
